# *Brucella suis* Infection in Dogs, Georgia, USA

**DOI:** 10.3201/eid1712.111127

**Published:** 2011-12

**Authors:** Sheela Ramamoorthy, Moges Woldemeskel, Alan Ligett, Ron Snider, Robert Cobb, Sreekumari Rajeev

**Affiliations:** University of Georgia, Tifton, Georgia, USA (S. Ramamoorthy, M. Woldemeskel, A. Ligett, R. Snider, S. Rajeev);; Georgia Department of Agriculture, Atlanta, Georgia (R. Cobb)

**Keywords:** brucellosis, Brucella suis, bacteria, infection, zoonosis, dogs, orchitis, Georgia

**To the Editor:** Brucellosis is a serious, globally distributed zoonotic disease. Humans are susceptible to infection by *Brucella suis*, *B*. *abortus*, *B*. *melitensis*, and *B*. *canis* and can have lifelong symptoms of undulating fever, enlarged lymph nodes, malaise, and arthritis (*1*). In 2009, the United States was officially classified free of *B*. *abortus*. All states except Texas are classified as stage III (free) for swine brucellosis caused by *B*. *suis* (*2*).

Consumption of unpasteurized dairy products confers the highest risk for brucellosis in disease-endemic areas (*3*). However, hunters and owners of hunting dogs are at high risk for transmission of brucellosis from wildlife. Sporadic transmission of *B*. *canis* from pet dogs to their owners has been reported (*4**–**6*). We describe a recent increase in *B. suis* detection in dogs in southern Georgia, USA, and caution the public about the potential for transmission to humans in contact with infected dogs and wild hogs.

Smooth *Brucella* spp. express the immunodominant O side chain on the lipopolysaccharide of their surface. Therefore, this side chain forms the antigenic basis of diagnostic tests, such as the card test. The *B*. *abortus* plate antigen (BAPA) test can detect smooth species. Because *B*. *canis* does not express the O side chain on its surface, serologic tests for *B. canis* differ from tests for *B*. *abortus*, *B*. *suis*, or *B*. *melitensis* (*7*). Therefore, *B*. *suis*–infected dogs are unlikely to have positive results for *B*. *canis* tests and vice versa.

During June 2010–July 2011, a total of 674 canine serum samples submitted by veterinarians servicing 207 kennels or pet owners in Georgia were tested by using the BAPA and card agglutination tests. Positive dogs were not detected until late March 2011. However, 9 dogs from 4 counties (Laurens, Worth, Tift, and Dougherty) were seropositive by BAPA and card agglutination tests. The same dogs were seronegative by *B*. *canis*–specific tube agglutination and agar gel immunodiffusion tests. Results indicated exposure to *B*. *abortus*, *B*. *suis*, or *B*. *melitensis* (*7*). Examination of case histories showed that all seropositive dogs had been recently exposed to feral swine during hunting expeditions, which led to a presumptive diagnosis of *B*. *suis* infection in the exposed dogs. All dogs were subsequently euthanized.

Testicles from 2 of the dogs were subjected to *Brucella* spp. culture. These dogs were a hunting dog and a pet dog owned by a hog hunter. Culture of testicles from both animals showed a *Brucella* sp., which was identified as *B*. *suis* by using conventional biochemical testing and sequencing of the 16S rRNA gene. Both isolates were destroyed <7 days after confirmation according to select agent guidelines. Histopathologic examination of testicular tissue from affected dogs showed severe necrotizing, suppurative to pyogranulomatous epididymitis and orchitis.

Although transmission of *B*. *suis* from dogs to humans has not been reported, *B*. *suis* is second only to *B*. *melitensis* in its pathogenicity to humans (*1*). Therefore, dogs exposed to feral hogs should be tested for *Brucella* sp. and monitored for clinical signs, while keeping in mind that sensitivity and specificity of *B*. *abortus*–specific tests is unknown. If a pet is infected with *B*. *canis*, a long course of antimicrobial drugs and spaying or neutering to prevent breeding is advisable but might not be completely effective.

Because *B*. *suis* is more pathogenic to humans than *B*. *canis*, and its mechanisms of pathogenesis in dogs can vary, the same recommendations might not be true for *B*. *suis*–infected dogs. Given the serious zoonotic implications of *B*. *suis* infections, euthanasia of the affected pet may be advocated by regulatory agencies and physicians treating exposed humans.

Little information, including that for pathogenesis or duration of bacteremia, is available for *B*. *suis* infections in dogs. Therefore, blood cultures might not reliably detect *B*. *suis*–infected dogs. For *B*. *canis* infections, only animals certified free of *Brucella* spp. by 2 consecutive serologic or blood culture tests conducted 4–6 weeks apart can be used as breeding stock in kennels. If an outbreak occurs in a kennel, all infected animals should be isolated and euthanized after showing positive test results. The premises should also be thoroughly disinfected before restocking (*8*).

Classification of the United States as free of swine brucellosis is based on surveillance of domestic swine populations (*2*). However, many states classify swine into 3 categories: domestic swine that have no contact with feral swine, transitional swine that might have contact with feral swine, and feral swine. If a domestic swine herd is infected with *B*. *suis*, many states then reclassify that herd as transitional. Therefore, classification of a state as free of swine brucellosis does not mean that transitional herds or infected feral swine do not exist. Our results indicate possible underestimation of the role of feral swine in the sylvatic transmission of *B*. *suis* (*9**,**10*). Future surveillance of feral swine populations in southern Georgia is warranted to determine the prevalence of *B*. *suis*.
